# Cognitive-behavioural therapy skill acquisition: impact on trajectories in depression symptoms and suicidal ideation: secondary analysis of a randomised controlled trial of cognitive behavioural therapy plus fluoxetine in young people experiencing depression

**DOI:** 10.1007/s00787-026-03032-4

**Published:** 2026-04-24

**Authors:** Sarah E. Hetrick, Michael Berk, Olivia M. Dean, Andrew M. Chanen, Patrick D. McGorry D. McGorry, Sue M. Cotton, Michelle Torok, Lisa Catania, Christopher G. Davey, Katrina Witt

**Affiliations:** 1 Centre of Youth Mental Health, The University of Melbourne, Melbourne, Victoria Australia; 2 Orygen, Melbourne, Victoria Australia; 3 Department of Psychological Medicine, School of Medicine, Faculty of Medical and Health Sciences, University of Auckland, Auckland, Private Bag 92019 New Zealand; 4 Florey Institute for Neuroscience and Mental Health, Melbourne, Victoria Australia; 5 Deakin University, IMPACT Institute, School of Medicine and Barwon Health, Geelong, Victoria Australia; 6 School of Psychological Sciences, Monash University, Melbourne, Victoria Australia; 7 Turner Institute of Brain and Mental Health, Melbourne, Victoria Australia; 8 Black Dog Institute, University of New South Wales, Sydney, New South Wales Australia; 9 Melbourne School of Psychological Sciences, The University of Melbourne, Melbourne, Victoria Australia; 10 Department of Psychiatry, The University of Melbourne, Melbourne, Victoria Australia

**Keywords:** CBT, Depression, Psychotherapy, Treatment, Youth, Early intervention

## Abstract

**Introduction:**

Cognitive Behavioural Therapy (CBT) is the primary evidence-based treatment for major depressive disorder, with behavioural activation and cognitive restructuring skill acquisition proposed as key mechanisms.

**Objective:**

The relationship between CBT skill acquisition and reductions in depressive symptoms and suicidal ideation in young people remains unclear.

**Methods:**

Data were drawn from the Youth Depression Alleviation–Combined (YoDA-C) trial, a randomised controlled trial comparing CBT with fluoxetine or placebo in 15–25-year-olds diagnosed with major depressive disorder. Group-based trajectory modelling identified patterns of skill acquisition over 12 weeks. Demographic and clinical predictors of skill development were examined. Generalised linear mixed models (GLMMs) assessed associations between skill trajectories and changes in Montgomery–Åsberg Depression Rating Scale (MADRS) and Suicidal Ideation Questionnaire (SIQ) scores.

**Results:**

Three trajectories emerged: Low-Stable (47.6%), Moderate-Improving (42.2%), and High-Improving (10.1%). The Low-Stable group showed minimal skill gains, while the Moderate- and High-Improving groups demonstrated moderate to substantial improvements. GLMMs revealed significant reductions in MADRS and SIQ scores across all groups; however, rates of symptom change did not differ significantly between trajectories.

**Conclusion:**

CBT skill acquisition trajectories were not associated with differential symptom improvement, suggesting symptomatic recovery may occur independently of skill mastery. These findings highlight the potential importance of broader therapeutic factors, such as the therapeutic alliance, in moderating outcomes for young people with major depression.

**Trial registration:**

Prospectively registered with the Australian New Zealand Clinical Trials Registry (ACTRN12612001281886).

## Introduction

Depression is the second leading cause of years lived with disability globally [1], affecting an estimated 332 million people in 2021 [1]. In Australia, one-in-five (21.3%) young people aged 10 to 24 years experience major depressive disorder [2]. Over the past three decades, the incidence, prevalence, and burden of major depression has increased both overall [1], and for young people specifically [3]. These trends were already evident prior to the declaration of the Coronavirus Disease 2019 (COVID-19) pandemic by the World Health Organization on 11 March 2020 [4], but have since worsened, with females [1] and young people [5] being disproportionately affected.

Cognitive behaviour therapy (CBT), either alone or in combination with adjunctive pharmacotherapy, is an effective first-line treatment for major depression [6], including for young people [7], and is recommended in clinical practice guidelines in Australia [8] and internationally [9–11]. CBT is a multicomponent and complex intervention, blending behavioural and cognitive therapy into an approach that seeks to explore and modify the relationship between thoughts, emotions, and behaviours. There is inconsistent evidence that CBT may be effective in reducing suicidal ideation [12], likely related to treatment protocols varying across trials. Some protocols specifically target suicidal ideation, whilst others adopt a broader focus.

Specific CBT components, such as cognitive restructuring and behavioural activation may mediate and explain variation in treatment effectiveness, although findings are mixed. One small study of three young people with major depression aged between 10 and 11 years found substantial individual variation in the impact of various CBT modules on depression symptom severity [13]. In a larger study of 282 young people aged between 10 and 20 years, CBT components were grouped into categories – cognitive restructuring, behavioural activation, problem solving and relaxation – with the relative ordering of these components randomised. This study found that delivering cognitive restructuring components earlier in the course of treatment may yield more rapid decreases in depression symptom severity [14]. Similar findings in adults highlight the potential for ‘sudden gains’ following cognitive restructuring [15].

Overall, however, little is known about which components of CBT may be most effective for youth depression [16]. Focusing on CBT skill acquisition — the skills developed through behavioural and cognitive restructuring techniques used in therapy — could offer valuable insights into individual differences in treatment response. Studies in adults suggest that CBT skill acquisition may be a critical mechanism of change [17, 18], though this has not yet been thoroughly explored in young people.

The Youth Depression Alleviation–Combined Treatment (YoDA-C) trial [19] offers a tailored dataset to investigate whether CBT skills acquisition is associated with treatment response in young people. In this trial, young people aged 15 to 25 years were randomised to CBT plus fluoxetine or CBT plus placebo over a 12-week treatment period. The CBT approach was manualised, and included both ‘core’ and ‘targeted’ modules delivered in a flexible and formulation-driven approach, in line with best practice [20]. The seven core modules consisted of behavioural and cognitive restructuring components that were delivered to all participants, and each could be revised and practised over several sessions. Individually ‘targeted’ modules were only introduced later in the 12-week treatment course where indicated [19]. While no statistically significant overall differences were found between groups, both showed similar improvements in depression and suicidal ideation symptom severity. Secondary analyses revealed distinct response patterns, highlighting the need for more targeted interventions earlier in the treatment course for those who did not achieve symptom relief [21].

Building on this, the aim of the present study was to explore variability in the acquisition of CBT skills during the YoDA-C trial, investigate whether certain demographic and/or clinical factors are differentially associated with membership in each identified CBT skills acquisition trajectory, and explore the relationship between CBT skills acquisition and both depression and suicidal ideation symptom resolution. This secondary analysis provides critical insights into the mechanisms of treatment response to CBT and may inform strategies to enhance treatment outcomes for youth with major depression and suicidal ideation.

## Methods

This study involved secondary analysis of the YoDA-C trial; a randomised, double-blind, placebo-controlled, multicentre clinical trial that was prospectively registered with the Australian and New Zealand Clinical Trials Register (ACTRN12612001281886). The study was approved by the Melbourne Health Human Research Ethics Committee (HREC/12/MH/151). Detailed methodological information on this trial is reported elsewhere [22].

### Participants and setting

Participants were recruited from one of four enhanced primary care community-based youth mental health treatment centres and one tertiary/specialist service located in western Melbourne, Australia.

Eligible participants were: (1) aged between 15 and 25 years; (2) diagnosed with major depressive disorder (MDD) according to the Structured Clinical Interview for the Diagnostic and Statistical Manual for mental disorders (DSM), Axis I Disorders (SCID-IV); (3) scored 20 or greater on the Montgomery-Åsberg Depression Rating Scale (MADRS) [23], indicating at least moderate levels of depression at baseline, and; (4) able to provide written informed consent, including both an adequate intellectual capacity and English language fluency.

Young people were excluded if they had: (1) a lifetime or current diagnosis of psychosis according to the SCID-I/P; (2) a lifetime or current diagnosis of bipolar I or II disorder; (3) a diagnosis of an acute or unstable medical disorder that would interfere with treatment; (4) a positive pregnancy screen; (5) a severe disturbance such that they would be unable to comply with the requirements of informed consent or with the study protocol (noting parents also gave consent for those aged 15–17); (6) currently received treatment with an antidepressant medication for at least two weeks prior, or; (7) previously been treated with fluoxetine that was either ineffective or poorly tolerated. Participants could not be treated with any antipsychotic medications throughout the duration of the trial (and if that was indicated, those participants would have been excluded), although treatment with benzodiazepines or benzodiazepine-like medications was permitted if clinically indicated.

### Randomisation and masking

Following screening, eligible participants were sequentially randomised to receive either CBT+fluoxetine or CBT+placebo on a 1:1 basis (see CONSORT diagram in the primary trial report) [22]. Further details on the allocation sequence and concealment processes used in the overall YoDA-C trial are reported elsewhere [19, 22].

### Intervention and control conditions

Young people allocated to the CBT+fluoxetine condition received a daily 20-mg tablet of fluoxetine. Dosage could be increased up to 40-mg if clinically indicated. Participants allocated to the CBT+placebo group received one pill identical in packaging, appearance, colour and taste to the trial medication.

All participants were concurrently offered weekly sessions of CBT for up to 12 weeks. The sessions covered seven core components of manualised CBT: (1) psychoeducation; (2) understanding and monitoring emotions; (3) behavioural activation; (4) chain analysis; (5) identifying automatic thoughts; (6) working with unhelpful thoughts, and (7) relapse prevention.

### Outcomes

A full description of measures (including baseline and covariates) in this trial is included in the trial protocol paper [22]. For this study, the primary outcome measure, depression symptom severity, was measured using the clinician-rated MADRS, a 10-item scale in which higher scores indicate greater depression severity.

Secondary outcomes included suicidal ideation and CBT skills acquisition. Suicidal ideation was measured using the Suicidal Ideation Questionnaire (SIQ) [24], a 30-item self-reported scale. Items are scored on a seven-point Likert scale ranging from zero to six with higher scores indicating greater suicidal ideation severity. A cut-point of 41 is used to indicate clinically significant suicidal ideation. We have previously ascertained the validity of this measure in young people with major depression [25].

CBT skill acquisition was measured by the Cognitive-Behavioural Therapy Skills Questionnaire (CBTSQ) [26]. This measure provides an overall score of general CBT skill acquisition as well as two subscale scores, one measuring acquisition of behavioural activation skills (Cognitive- Behavioural Therapy Skills Questionnaire—Behavioural Activation; CBTSQ-BA) and one measuring acquisition of cognitive restructuring skills (Cognitive-Behavioural Therapy Skills Questionnaire—Cognitive Restructuring; CBTSQ-CR). Higher scores indicate greater skill acquisition.

Data on these outcomes were collected at baseline, and again at weeks 4, 8, and 12, with a further follow-up telephone assessment of the MADRS at 26 weeks.

### Statistical analysis

We have previously used latent group trajectory modelling to the define trajectories of change in depression and suicidal ideation severity over the course of the 12-week YoDA-C trial, and to characterise the factors associated with membership to these trajectories [21]. In this study, we use the same approach to define trajectories of CBT skills acquisition.

Following our previous approach, trajectory enumeration was assessed using the recommended two stage approach, first, by defining the optimal number of trajectories, and then, determining the polynomial shape of each [27]. Model fit was guided by the magnitude of the sample size-adjusted Bayesian Information Criterion (BIC), the Odds of Correct Classification (OCC), and model entropy. We also ensured the average posterior probability for each group exceeded 0.80, and each trajectory had significant parameter estimates (*p* < 0.05) [28]. Models were weighted by the inverse of the probability of trajectory membership derived from the posterior probability estimates. All analyses were conducted in Stata for Windows, version 13, using the *traj* plug-in [29].

Multinomial logistic regression models were used to investigate associations between demographic and/or clinical factors and membership in each CBT skills acquisition trajectory. Demographic covariates, treated as time-invariant, included: sex (with male sex as the reference category), age (with those over 18 years at baseline as the reference category due to observed differences in treatment response for older participants) [19], and treatment allocation (with allocation to CBT+fluoxetine as the reference category).

Given that clinical severity and comorbidity may influence CBT skills acquisition, several baseline covariates were also included: number of previous depression episodes, total scores on the Alcohol Use Disorders Identification Test (AUDIT) [30], the Generalised Anxiety Disorders – 7-item (GAD-7) [31], and the Social and Occupational Functioning Scale (SOFAS) [32]; past month history of attempted suicide and/or self-harm assessed using the Columbia Suicide Severity Rating Scale (C-SSRS) [33]; and four personality factors from the Dimensional Assessment of Personality Pathology-Basic Questionnaire (DAPP-BQ) [34] – emotion dysregulation, dissocial behaviour, social avoidance, and compulsiveness.

Finally, post-hoc Generalized Linear Mixed-effects Models (GLMMs) were used to examine the relationship between time and total scores on both the MADRS and SIQ throughout the treatment period, as a function of CBT skills acquisition trajectories. The models were fit using a Gaussian family with an identity link function. These analyses were conducted in Stata for Windows, version 13, using the *meglm* package.

### Role of the funding source

Funders had no role in study design, data collection, analysis, interpretation, or in writing of the manuscript.

## Results

A total of 153 young people aged 15 to 25 years (mean: 19.6 ± 2.7 years) diagnosed with major depressive disorder were randomly assigned to receive either CBT+fluoxetine (*N =* 76) or CBT+placebo (*N* = 77). Further details on characteristics of the participants at baseline are reported elsewhere [19, 21, 22]. In brief, most were female (*n =* 91; 58.7%), aged over 18 years at baseline (*n =* 116; 74.8%), and had moderate to severe levels of depression at baseline according to established cut-off scores for the MADRS (mean: 32.9 ± 5.4). The median number of depressive episodes at baseline was 2.0 (interquartile range: 1.0–4.0). Over one-third (*n* = 61; 39.9%) were experiencing their first episode of depression.

Three participants did not have sufficient follow-up data to enable trajectory enumeration. The following analyses are therefore based on data for the remaining participants.

### CBT skills acquisition trajectories

For both the behavioural activation and cognitive restructuring subscales of the CBTSQ, a three-trajectory model provided optimal fit (Table 1). Trajectories were defined by linear polynomials indicating that participants’ acquisition of skills in both behavioural activation and cognitive restructuring improved steadily across each assessment time point.

**Table 1:** Latent trajectory fit statistics for the behavioural activation and cognitive restructuring subscales of the CBTSQ

CBTSQ Subscale	Model	Sample Size Adjusted BIC	Log Bayes Factor	Model Entropy	OCC				Average Posterior Probabilities				Number of Participants			
					1	2	3	4	1	2	3	4	1	2	3	4
Behavioural activation																
	1	-1610.51	-	-	0.99	-	-	-	1.00	-	-	-	153	-	-	-
	2	-1557.76	-26.38	0.72	4.00	21.51	-	-	0.92	0.87	-	-	117	38	-	-
	3	-1542.17	-7.79	0.71	10.35	5.22	116.18	-	0.87	0.85	0.93	-	60	80	15	-
	4	-1542.17	3.62	0.64	16.08	2.94	14.07	72.50	0.82	0.75	0.76	0.87	35	78	29	13
Cognitive restructuring																
	1	-1803.00	-	-	0.99	-	-	-	1.00	-	-	-	153	-	-	-
	2	-1720.06	-41.47	0.84	8.69	42.00	-	-	0.97	0.93	-	-	119	36	-	-
	3	-1700.79	-9.63	0.75	11.75	6.46	116.46	-	0.89	0.85	0.95	-	62	71	22	-
	4	-1706.04	2.63	0.75	11.75	5.82	49.71	57.56	0.86	0.83	0.93	0.97	54	71	14	16

*BIC* Bayesian Information Criterion, *CBTSQ* Cognitive Behavioural Therapy Skills Questionnaire, *OCC* Odds of Correct Classification. Model number refers to the number of trajectories modelled

### Behavioural activation

**Fig. 1:**
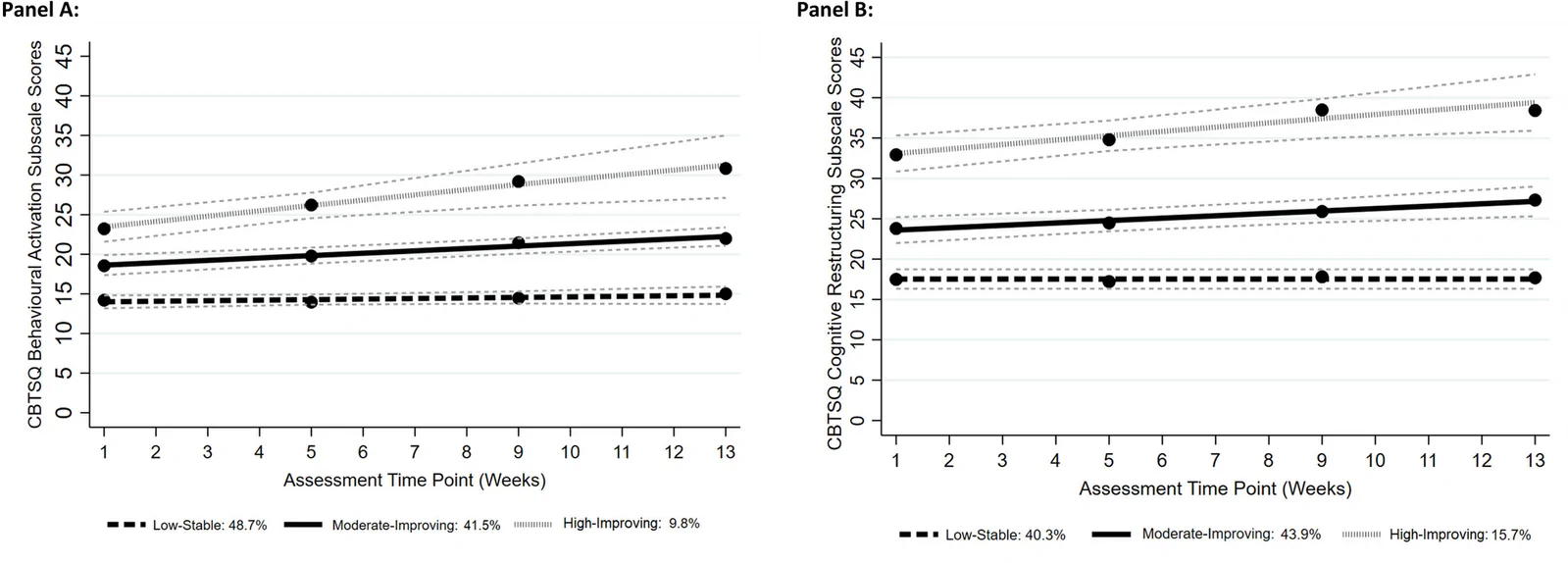
Three-group averaged trajectory model for scores on the CBTSQ Behavioural Activation Subscale (Panel **A**) and Cognitive Restructuring Subscale (Panel **B**) across assessment time points (weeks)

**Fig. 2:**
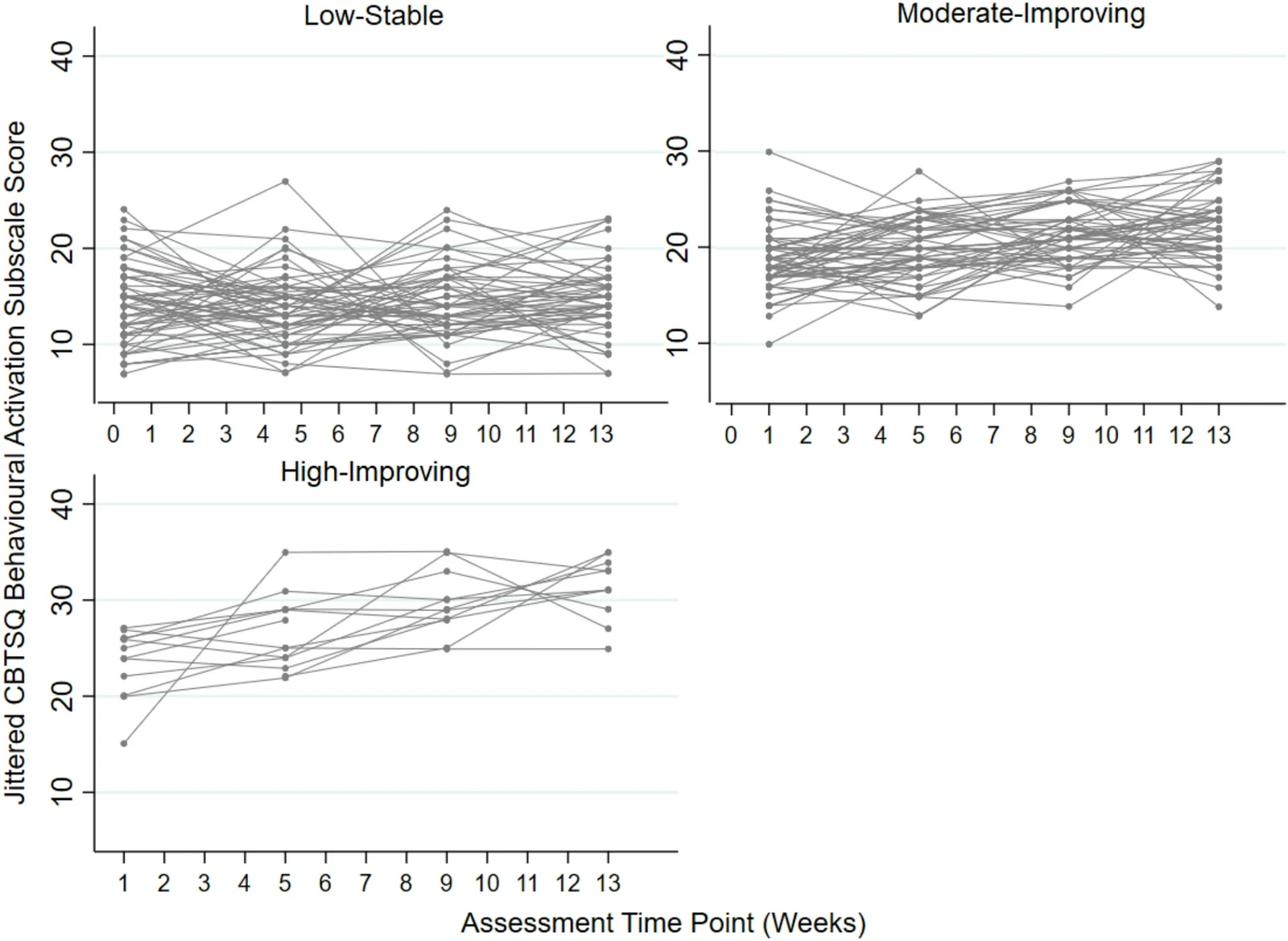
Individualised trajectories for scores on the CBTSQ Behavioural Activation Subscale across assessment time points (weeks)

For CBTSQ-BA, the first trajectory (i.e., Low-Stable) accounted for just under half (48.7%) of the sample. Participants in this trajectory group had relatively low levels of behavioural activation skills at baseline (mean: 14.5 ± 4.1, range: 7–24), which did not improve significantly by the end of treatment at 12 weeks (mean: 15.3 ± 3.7, range: 7–23; paired *t* = -0.52, *df* = 85, *p* = 0.603). The second trajectory (i.e., Moderate-Improving) accounted for 41.5% of the sample. Participants in this trajectory group had moderate levels of behavioural activation skills at baseline (mean: 19.4 ± 3.3, range: 13–30), which improved by the end of treatment (mean: 22.4 ± 3.2, range: 14–29; paired *t* = -5.27, *df* = 73, *p* < 0.001). The final trajectory (i.e., High-Improving) accounted for around one-in-ten (9.8%) and consisted of those with high levels of behavioural activation skills at baseline (mean: 22.9 ± 3.9, range: 15–27), which also improved significantly by end of treatment (mean: 31.6 ± 2.6, range: 27–35; paired *t* = -10.99, *df* = 16, *p* < 0.001). See Fig. 1, Panel A for averaged trajectory plots, and Fig. 2 for individualised trajectory plots.

Compared to those assigned to the Low-Stable trajectory group for behavioural activation skills acquisition, young people assigned to the Moderate-Improving trajectory group had higher scores on the SOFAS at baseline. Those assigned to the High-Improving trajectory group had lower scores on the Social Avoidance Cluster of the DAPP-BQ, measured at baseline as compared to those assigned to the Low-Stable trajectory group. No other demographic or clinical characteristic, including treatment assignment (i.e., CBT+fluoxetine vs. CBT+placebo), were associated with membership to the CBTSQ-BA trajectories (Table 2).

**Table 2:** Univariate logistic regression models predicting membership to CBTSQ trajectory groups

CBTSQ Subscale	Covariates (Measured at baseline)		Trajectory							
			Low-Stable		Moderate-Improving			High-Improving		
CBTSQ-BA		RRR	95% CI	*p*	RRR	95% CI	*p*	RRR	95% CI	*p*
	Sex - male		(Reference)		1.52	(0.77–3.00)	0.224	1.52	(0.46–5.00)	0.487
	Age - over 18 years		(Reference)		1.13	(0.55–2.29)	0.746	1.13	(0.31–4.01)	0.856
	Treatment allocation – CBT+Fluoxetine		(Reference)		1.25	(0.64–2.44)	0.506	1.33	(0.41–4.34)	0.633
	Number previous depressive episodes		(Reference)		1.23	(0.98–1.54)	0.071	1.12	(0.78–1.63)	0.536
	Previous psychiatric treatment		(Reference)		0.69	(0.37–1.29)	0.247	1.42	(0.50–4.02)	0.506
	Total AUDIT scores		(Reference)		0.98	(0.90–1.04)	0.345	1.09	(0.99–1.19)	0.084
	Total GAD-7 scores		(Reference)		1.06	(0.99–1.13)	0.069	1.03	(0.92–1.16)	0.553
	Past month self-harm		(Reference)		0.88	(0.43–1.81)	0.723	0.39	(0.08–1.88)	0.239
	DAPP-BQ emotional dysregulation cluster		(Reference)		1.00	(0.99–1.01)	0.456	0.99	(0.98–1.00)	0.071
	DAPP-BQ dissociative personality cluster		(Reference)		1.01	(0.99–1.02)	0.052	0.99	(0.98–1.02)	0.727
	DAPP-BQ socially avoidant personality cluster		(Reference)		0.98	(0.97–1.00)	0.119	**0.93**	**(0.89–0.97)**	**0.001**
	DAPP-BQ obsessive-compulsive personality cluster		(Reference)		1.04	(0.99–1.08)	0.113	1.04	(0.97–1.11)	0.268
	Total SOFAS scores		(Reference)		**1.04**	**(1.01–1.07)**	**0.005**	1.04	(0.96–1.09)	0.081
CBTSQ-CR										
	Sex - male		(Reference)		1.19	(0.60–2.36)	0.618	2.67	(0.80–8.90)	0.111
	Age - over 18 years		(Reference)		0.91	(0.44–1.86)	0.794	1.02	(0.29–3.64)	0.972
	Treatment allocation – CBT+Fluoxetine		(Reference)		1.33	(0.68–2.60)	0.407	1.36	(0.42–4.39)	0.612
	Number previous depressive episodes		(Reference)		1.02	(0.84–1.25)	0.829	0.96	(0.64–1.40)	0.815
	Previous psychiatric treatment		(Reference)		0.58	(0.30–1.09)	0.088	1.26	(0.47–3.38)	0.649
	Total AUDIT scores		(Reference)		0.96	(0.90–1.03)	0.232	1.02	(0.93–1.13)	0.645
	Total GAD-7 scores		(Reference)		1.00	(0.94–1.06)	0.982	1.04	(0.93–1.16)	0.514
	Past month self-harm		(Reference)		1.02	(0.50–2.10)	0.958	0.18	(0.02–1.49)	0.112
	DAPP-BQ emotional dysregulation cluster		(Reference)		0.99	(0.98–1.00)	0.363	**0.98**	**(0.97–0.99)**	**0.022**
	DAPP-BQ dissociative personality cluster		(Reference)		1.00	(0.99–1.01)	0.704	0.99	(0.96–1.01)	0.206
	DAPP-BQ socially avoidant personality cluster		(Reference)		**0.98**	**(0.96–0.99)**	**0.024**	**0.93**	**(0.90–0.97)**	**0.001**
	DAPP-BQ obsessive-compulsive personality cluster		(Reference)		1.00	(0.96–1.04)	0.854	1.06	(0.98–1.14)	0.147
	Total SOFAS scores		(Reference)		**1.05**	**(1.02–1.08)**	**0.001**	1.03	(0.99–1.07)	0.167

*AUDIT* Alcohol Use Disorders Identification Test, *CBTSQ* Cognitive Behavioural Therapy Skills Questionnaire, *CBTSQ-BA* Cognitive Behavioural Therapy Skills Questionnaire, Behavioural Activation Subscale, *CBTSQ-CR* Cognitive Behavioural Therapy Skills Questionnaire, Cognitive Restructuring Subscale, *CI* Confidence interval, *DAPP-BQ* Dimesional Assessment of Personality Pathology – Basic Questionnaire, *GAD-7* Generalised Anxiety Disorders – 7, *MADRS* Montgomery-Åsberg Depression Rating Scale, *p* Probability value, *RRR* Relative risk ratio, *SOFAS* Social and Occupational Functioning Assessment Scale. Boldface indicates covariates significant at the conventional p<0.05 level. Dashes indicate insufficient numbers to enable calculation.

### Cognitive restructuring

**Fig. 3:**
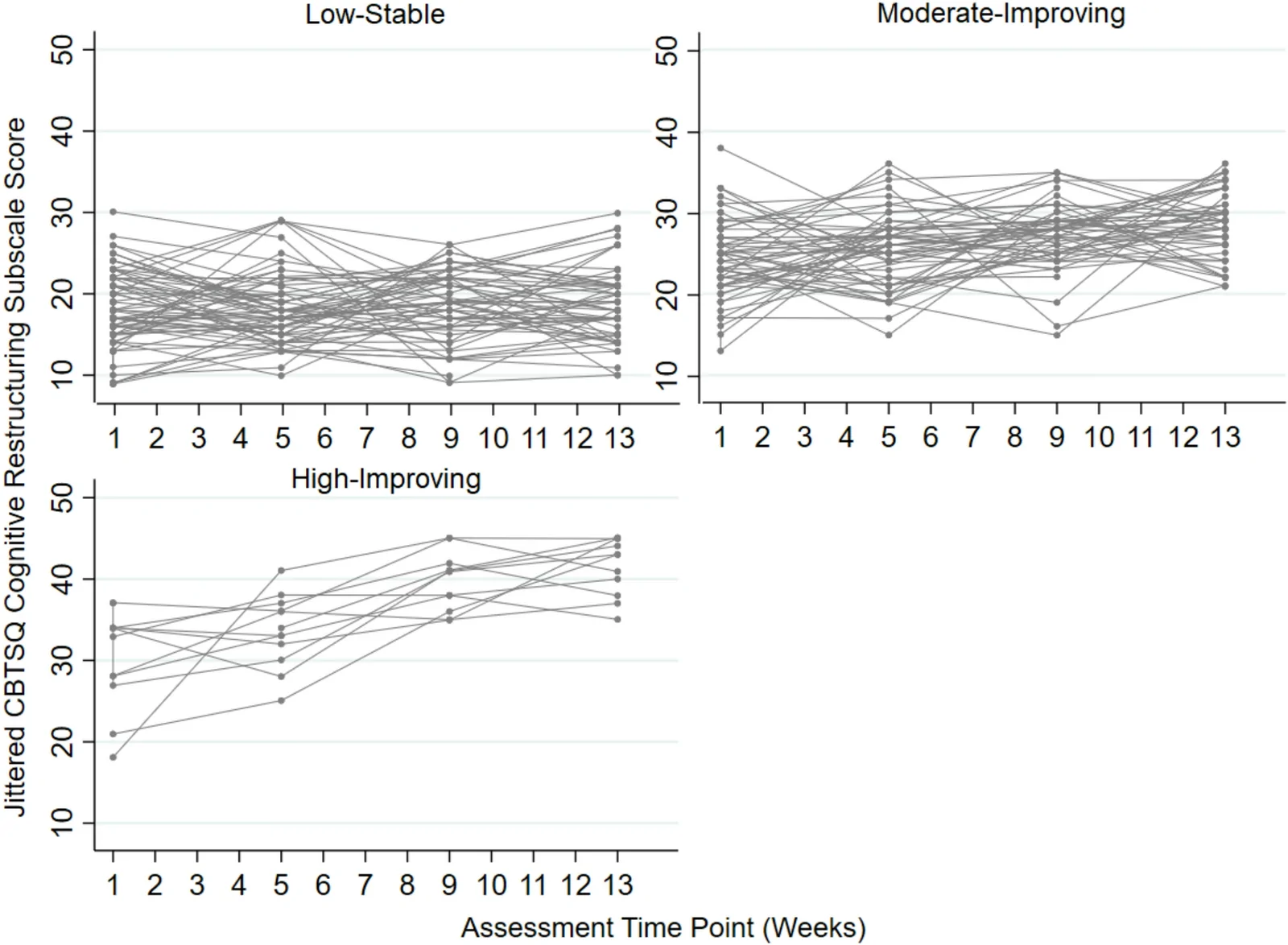
Individualised trajectories for scores on the CBTSQ Cognitive Restructuring Subscale across assessment time points (weeks)

For CBTSQ-CR, the first trajectory (i.e., Low-Stable) accounted for 40.3% of the sample. Young people in this group had relatively low levels of cognitive restructuring skills at baseline (mean: 18.2 ± 4.5, range: 9–30), which did not improve significantly by the end of treatment (mean: 18.5 ± 4.3, range: 10–30; paired *t* = 0.24, *df* = 78, *p* = 0.809). The second trajectory (i.e., Moderate-Improving) accounted for 43.9% of the sample. Participants in this trajectory group had moderate levels of cognitive restructuring skills at baseline (mean: 24.4 ± 4.4, range: 13–38), which improved by the end of treatment (mean: 28.6 ± 4.0, range: 21–36; paired *t =* -5.97, *df* = 78, *p* < 0.001). The final trajectory (i.e., High-Improving) accounted for 15.7% of the sample and consisted of those with high levels of cognitive restructuring skills at baseline (mean: 30.0 ± 6.4, range: 18–37), which improved significantly by end of treatment (mean: 41.7 ± 3.5, range: 35–45; paired *t* = -8.44, *df* = 18, *p* < 0.001). See Fig. 1, Panel B for averaged trajectory plots, and Fig. 3 for individualised trajectory plots.

Compared to those assigned to the Low-Stable trajectory group for cognitive restructuring skills acquisition, young people assigned to the Moderate-Stable trajectory group had lower scores on the Social Avoidance Cluster of the DAPP-BQ and higher total SOFAS scores at baseline. Those assigned to the High-Improving trajectory group had lower scores on both the Emotional Dysregulation and Socially Avoidant Clusters of the DAPP-BQ at baseline compared to those assigned to the Low-Stable trajectory group. Again, treatment assignment (i.e., CBT+fluoxetine vs. CBT+placebo) was not associated with cognitive restructuring trajectory assignment (Table 2).

### Impact of CBT skills acquisition trajectories on depression severity

While MADRS scores showed a significant reduction over time, particularly evident in weeks 4 and 12, there were no significant differences between CBTSQ-BA or CBTSQ-CR trajectory groups (Table 3).

**Table 3:** Generalised linear mixed-effects models of MADRS and SIQ scores as a function of CBTSQ-BA and CBTSQ-CR trajectory group membership

Predictors		MADRS Total Scores			SIQ Total Scores		
		β	SE	*p*			
Time							
	*Baseline (reference)*	-	-	-	-	-	-
	*Week 4*	**0.12**	**0.05**	**0.039**	**0.49**	**0.09**	**< 0.001**
	*Week 8*	-0.10	0.06	0.088	**0.32**	**0.09**	**0.001**
	*Week 12*	**0.20**	**0.05**	**< 0.001**	0.04	0.09	0.634
	*Week 26*	0.06	0.04	0.134	-	-	-
CBTSQ-BA trajectories							
	*Low-Stable (reference)*	-	-	-	-	-	-
	*Moderate-Improving*	-0.06	0.91	0.950	3.27	4.89	0.504
	*High-Improving*	2.08	1.44	0.149	7.40	6.97	0.288
CBTSQ-CR trajectories							
	*Low-Stable (reference)*	-	-	-	-	-	-
	*Moderate-Improving*	-1.19	0.88	0.178	1.21	4.71	0.797
	*High-Improving*	1.55	1.40	0.269	5.30	7.03	0.451

*CBTSQ-BA* Cognitive Behavioural Therapy Skills Questionnaire – Behavioural Activation subscale, *CBTSQ-CR* Cognitive Behavioural Therapy Skills Questionnaire – Cognitive Restructuring subscale, *MADRS* Montgomery-Åsberg Depression Rating Scale, *p* Probability value, *SE* Standard error, *SIQ* Suicidal Ideation Questionnaire. Boldface indicates covariates significant at the conventional *p* < 0.05 level. Dashes indicate insufficient numbers to enable calculation

### Impact of CBT skills acquisition trajectories on suicidal ideation severity

Similarly, whilst SIQ scores demonstrated significant reductions over time, particularly evident in weeks 4 and 8, again there were no significant differences between CBTSQ-BA or CBTSQ-CR trajectory groups (Table 3).

## Discussion

CBT skill acquisition—particularly behavioural activation and cognitive restructuring skills—has been proposed as a key factor underlying symptomatic improvement following CBT for major depression. However, research examining how CBT skill acquisition trajectories may relate to symptomatic change over the course of treatment is limited, particularly in young people. Using secondary data from the Youth Depression Alleviation–Combined (YoDA-C) trial, a randomised, double-blind, placebo-controlled trial of Cognitive Behavioural Therapy (CBT) plus fluoxetine or placebo in young people with major depression [19], we identified three distinct trajectories of self-rated CBT skill acquisition over the 12-week treatment period. These findings align with previous work on internet-delivered CBT (iCBT) in adults with major depression [35]. Approximately half the sample had persistently low levels of behavioural activation and cognitive restructuring across treatment. Just under half had moderate skill levels at baseline, which improved over time, while one-in-ten began with high skill levels and also showed significant improvement. These results underscore substantial individual variability in CBT skill acquisition during treatment.

Contrary to clinical guidelines that recommend combined psychological treatment and pharmacotherapy as the first-line treatment for major depression in Australia [8] and internationally [9–11], we found that treatment allocation did not affect CBT skill acquisition. This aligns with some adult studies suggesting that adding adjunct antidepressant medications may not directly enhance CBT-specific skill development [36]. However, the possibility of a bidirectional interaction should be explored. Taken together, these findings highlight the need to investigate how different CBT treatment components interact positively and negatively.

Factors theorised to influence CBT skill acquisition—such as age (as a proxy for cognitive, emotional, and social maturity) [16], sex/gender [37], previous depressive episodes (as a proxy for cognitive flexibility) [38], and previous psychiatric treatment (as a proxy for exposure to psychotherapeutic techniques) – were not associated with trajectory group membership in this study. However, certain personality traits and social functioning were significantly associated with trajectory group membership. For behavioural activation skill acquisition, young people in the Moderate-Stable group had higher social functioning scores compared to those in the Low-Stable group, and those assigned to the High-Improving group had lower social avoidance. For cognitive restructuring skill acquisition, those in the Moderate-Improving had higher social functioning and lower social avoidance as compared to those in the Low-Stable group, while those in the High-Improving group had lower emotional dysregulation and social avoidance. Taken together, these results highlight the complex interplay of social, emotional, and personality factors in skill CBT skill acquisition and highlights the potential value of adjunctive supports targeting emotional regulation and self-efficacy.

While there is a theoretical understanding of how CBT works, the evidence regarding the mechanisms of change are underdeveloped at present [39]. Although prior work has linked skill acquisition, particularly cognitive restructuring, to reductions in depression symptom severity in adults [17, 18], this study did not find similar evidence in young people. We found no evidence that trajectories of CBT skill acquisition, either for the behavioural activation or cognitive restructuring subscale, was significantly associated with changes in depression or suicidal ideation severity over the 12-week treatment period. While depression severity improved significantly by week 12 and suicidal ideation severity declined between weeks 4 and 8, these improvements were independent of CBT skill acquisition groups. These findings suggest that therapeutic improvements in depression and suicidal ideation may be driven by broader therapeutic factors, such as the therapeutic alliance, treatment engagement, or nonspecific psychotherapy effects. This is supported by meta-analyses comparing different psychotherapeutic techniques, many of which conclude that these approaches appear to be equally effective [40].

It is also possible that the timing of skill acquisition and symptom improvement differs for young people as compared with adults. This highlights the complexity of CBT mechanisms of change in young people with major depression who may require additional support to apply skills learnt outside the structured context of therapy. Future research could explore whether incorporating more frequent sessions, extending treatment duration, or providing explicit opportunities to practice skills outside therapy sessions enhances the association between CBT skills acquisition and symptom improvement.

### Limitations

We were unable to measure CBT skills use outside treatment sessions, a limitation common in clinical trials [41]. CBT skills were measured via the self-report CBT-SQ so that confounding with depression recovery (or lack of) may account for the lack of relationship between depression improvement and skill acquisition. Baseline assessments of pre-treatment expectations, cognitive ability, therapeutic alliance, and nonspecific treatment effects — as well as initial treatment effects like sleepiness, which are known to influence CBT engagement [42] — were also not assessed for this study. Additionally, CBT was delivered by therapists from diverse professional backgrounds, including Masters-level or higher clinical psychologists, occupational therapists, and social workers, potentially leading to differences in emphasis on behavioural activation versus cognitive restructuring. However, prior research suggests therapist type may have minimal impact on CBT skills acquisition [43].

It is important to note that for both behavioural activation and cognitive restructuring skills acquisition, the High-Improving trajectories comprised a small proportion of the sample (9.8% and 15.7%, respectively). Accordingly, these findings should be interpreted cautiously and as characterising observed patterns within this cohort, with replication in larger samples needed to assess their stability.

### Clinical implications

Our findings highlight the need for tailored CBT delivery. Young people struggling to acquire core CBT skills may benefit from supplementary interventions such as emotion regulation training or extended therapeutic engagement. Future trials should include mechanisms-of-change measures, such as the impact of CBT skill acquisition on negative thinking and cognitive restructuring.

## Conclusions

We identified three distinct trajectories of skill acquisition for both behavioural activation and cognitive restructuring following 12 weeks of CBT for young people with major depression. About half of the young people were assigned to either Low-Stable or Moderate-Improving trajectories for both skills, while only around one-in-ten were assigned to the High-Improving trajectories. Despite these distinct trajectories, depression and suicidal ideation severity did not significantly differ across CBT skill acquisition groups throughout the treatment period. These findings suggest that the therapeutic effect of CBT may not be directly linked to the rate or extent of CBT skill acquisition as measured by behavioural activation and cognitive restructuring. Instead, broader therapeutic factors, such as the therapeutic alliance, treatment engagement, or other nonspecific factors of psychotherapy, may play a more central role in moderating clinical improvements in depression and suicidal ideation severity. Future research should explore how these common factors interact with skill acquisition processes to influence treatment outcomes, providing a more nuanced understanding of the mechanisms of action of CBT for in young people with major depression.

## Data Availability

The de-identified participant data that underlie the results reported in this manuscript, along with a data dictionary, the study protocol, and the participant information and consent form, will be made available to researchers following approved by the YoDA-C investigators. Proposals should be directed to [c.davey@unimelb.edu.au](mailto: c.davey@unimelb.edu.au) .
